# Flehmen, Osteophagia, and Other Behaviors of Giraffes (*Giraffa giraffa angolensis*): Vomeronasal Organ Adaptation

**DOI:** 10.3390/ani13030354

**Published:** 2023-01-19

**Authors:** Lynette A. Hart, Benjamin L. Hart

**Affiliations:** 1 Department of Population Health and Reproduction, School of Veterinary Medicine, University of California, Davis, CA 95616, USA; 2 Department of Anatomy, Physiology and Cell Biology, School of Veterinary Medicine, University of California, Davis, CA 95616, USA

**Keywords:** bone-chewing, estrus, nasopalatine duct, incisive papillae, osteophagia, reproductive behavior

## Abstract

**Simple Summary:**

Adult male giraffes (*Giraffa giraffa angolensis*) are far larger than the females; they also carry a sizable head and neck. Observations were made at the Namutoni waterholes, Etosha National Park in Namibia, to monitor males testing the females for sexual receptivity. Lacking estrous signals, males provoke females to urinate by sniffing and prodding them. If the female is going to urinate, she first widens her hindleg stance and sets a stable posture, and then urinates. The male gathers the urine in his mouth with his tongue and then frequently performs flehmen to assess her estrous status. The giraffe is unusual in never investigating urine on the ground. Giraffes have papillae on their hard palate that each open to a nasopalatine duct that enters the vomeronasal organ (VNO), which is used for pheromone assessment. Unusually, giraffes were found to lack a prominent nasal connection to the VNO. Many giraffes also spent extensive time periods seeking bones, chewing them, and some got into trouble from having bones lodged in their mouth. A dark giraffe was apparently killed by two lions, after which a steady procession of giraffes arrived for some days to investigate the carcass. A dark giraffe bull emitted loud, pulsed warning calls that resulted in the giraffes fleeing the area.

**Abstract:**

The size of adult male giraffes (*Giraffa giraffa angolensis*) far exceeds the size of the females. At the Namutoni waterholes in Etosha National Park, bulls were seen many times each day screening adult females for their pending sexual receptivity by provoking them to urinate; this mainly involved sniffing their genitalia. If the female accedes to the male’s invitation, she widens her hindleg stance, braces her body, and then urinates, usually for at least five seconds. The male places his muzzle and tongue in the urine stream, and then performs flehmen, often raising his head high in the air. Males never investigated urine on the ground. The bilateral papillae on the giraffe’s hard palate connect with the nasopalatine ducts, which enter the bilateral vomeronasal organ (VNO). Unlike many mammals, the giraffe’s VNO lacks a prominent connection to the nasal cavity and its connections to the oral cavity are primarily via the incisive papillae. Most days, some giraffes were observed searching for bones for extended periods, chewing them, and sometimes being troubled by a bone stuck in their mouth. A giraffe killed by lions was frequented for several days by a procession of giraffes investigating the carcass. A very dark-colored bull giraffe emitted loud pulsed growls that drove off most of the surrounding giraffes.

## 1. Introduction

African male artiodactyl ungulates commonly investigate the urine of females during the breeding season. This gives males an opportunity to detect chemosensory cues in a female’s urine and then learn the sexual status of the female being investigated. The chemical cues in urine in the proestrus stage may announce that the female will come into full estrus soon, making it worthwhile for the male to follow the female. The typical behavior of a male antelope, such as a gazelle or impala, when pursuing females is to come into contact with freshly eliminated urine, often on the ground, and sample the urine in his oral cavity. Males may then perform the lip-curling flehmen response to analyze the estrous status of the female, which may indicate if she is in proestrus. If estrus is approaching, the male may then guard the female until mating is accomplished. If estrus is not imminent, the male will walk away.

In the case of giraffes, the general details of the giraffe bull’s investigation of the female and the performance of flehmen and, sometimes, the subsequent mating were described by Cynthia Moss [1] in her classic book, “Portraits in the Wild”. This account drew upon the observations of Ann Innis/Ann Dagg [2,3], who continued by describing the bull gently sniffing, nudging, and/or kicking (laufschlag) the female, thereby inducing her to urinate [4,5]. Then, the male would collect the urine in his mouth, raise his head, and curl his lip in a flehmen response, sometimes then ejecting some urine from his mouth in a thin stream. Bercovitch and colleagues [6] assessed how the reproductive behavior varies with the reproductive cycle of the female, documenting that males were more likely to associate with, and sexually investigate, females when they were cycling than when they were either pregnant or acyclic.

Like the vast majority of mammalian species, male giraffes are polygynous, compete for females, and invest little in their offspring. Giraffe males exhibit extreme sexual size dimorphism, and large-necked males gain favored access to estrous females [7]. The male’s body weight is more than 50% greater than that of the female, and his head and neck continue growing, along with added bone; males have heightened risks of predation [8]. They are challenged by the difficulty of balancing a cantilevered neck and head upon a relatively slight body [9]. The mature male also becomes a very dark brown, providing an honest signal of strength and dominance: this is associated with greater mating success [10]. These dark males may even have a musth period [11].

Considering the problem of males who need to locate females that provide no prominent signals of when they are in estrus, research attention has focused on the vomeronasal organ (VNO). Moulton’s [12] description in 1967 mentioned that in mammals, the VNO used in reproduction opens either anteriorly, into the nasal cavity, or into the buccal cavity by way of the nasopalatine canal, with the VNO sensory epithelium connecting to the accessory olfactory bulb by the vomeronasal nerve. Estes, writing in 1972 [13], expanded our understanding of its role in mammalian reproduction and provided a table (adapted from Schilling 1970) charting the arrangements of the vomeronasal and incisive ducts in different groups of mammals. The table showed most artiodactyls, but not suids, as having openings of the vomeronasal ducts in both the mouth and nose. Giraffes were listed with a question mark, suggested as perhaps having a VNO communication with the nose only.

As is common in artiodactyls, a prominent funnel leads from the nasal cavity into the incisive duct that opens to the VNO and continues on to the incisive papilla. The flehmen response facilitates sucking material from the incisive papilla into the VNO. Bulls use their tongues to direct the urine being tested toward the VNO [14] and dogs have also been shown to actively use their tongues [15]. In giraffes, the tongue plays an active role in gathering urine from the urine stream; perhaps, male giraffes may also push the females’ urine into the incisive papillae.

Exactly how the urine is assessed has been investigated somewhat in several African antelopes that have bilateral incisive papillae located on the hard palate, just behind the dental pad; each papilla connects to an incisive duct that leads to the bilateral tubular VNO. The incisive papillae and ducts are involved in transferring fluid-borne material from the oral cavity to the VNO, the bilateral accessory olfactory organs that are specialized for detecting the chemical cues related to proestrus and estrus; these urinary cues are sex pheromones. Flehmen behavior facilitates the transport of the collected urine from the oral cavity into the VNO in numerous artiodactyls. This widespread behavior is manifested in many mammals, including horses, cows, cats, and deer. As shown in a study on goats, the occurrence of flehmen stimulates emptying the VNO and pulling the new material into the VNO from the oral cavity through the incisive duct [16]. The oral connection leads to the VNO, which is used by the male for the analysis of estrous pheromones in the female’s urine.

In giraffes, the vomeronasal organ includes sensory vomeronasal epithelia covering the medial region of the lumen and cartilage surrounds the soft tissue; veins likely play a role in pumping odorants into the lumen, and the giraffe have distinctive spongy small veins and capillaries that may serve as a secondary pump specific to giraffes [17].

In most ungulates, there is a nasal connection to the VNO as well as an oral connection, and it has been proposed as a possibility that the nasal route to the VNO may be used for assessing certain plant-oriented chemicals related to detecting the season [18]. The giraffe is the only ungulate species with a gestation period longer than a year. By breeding throughout the year, with some breeding peaks associated with rainfall, giraffes do not need to focus intently on a particular season.

The typical behavior of a male antelope, such as a gazelle or impala, when pursuing receptive females is to contact freshly eliminated urine, often on the ground, and sample the urine in his oral cavity [19]. The eland usually acquires urine by intercepting the stream as the female urinates, but they do sometimes sample urine on the ground. After acquiring the female’s urine, a male may then perform the lip-curling flehmen motion to analyze the estrous status of the female, to find if she is in proestrus. If estrus is approaching soon, the male may then guard the female until mating is accomplished. If estrus is not imminent, the male will walk away.

An interesting exception to this pattern was found in our previous work on certain alcelaphine antelopes, including topi, hartebeest, and wildebeest, which lack an incisive papilla and oral connection to the VNO [20]. Topi and hartebeest were also found to lack interest in female urine and failed to perform flehmen. Thus, these antelopes manifest a different behavior and anatomy than the other antelopes studied.

With our observations at waterholes during the dry season, giraffes sometimes congregated in large numbers and exhibited unusual behaviors. Bone chewing (osteophagia) was a behavior observed in multiple animals for extended periods. A description by Hutson and colleagues describes the typical behavior: “The bone was partly hidden inside the giraffe’s mouth and partly projecting forward from one side of the mouth, while the giraffe’s head was held high, and the lower jaws moved laterally. Profuse saliva dripped from the animal’s lips” [21] (p. 4140). Until now, this perhaps appeared to be an infrequent behavior; we sought to note how often this behavior was recorded during our field observations. Little is known about osteophagia in giraffes, but in crested porcupines, lactating females engage in this behavior, presumably to bolster their calcium and phosphorus levels [22].

The attentive responses of giraffes to a giraffe carcass are not described in the research literature: this behavior is first reported here. The high-amplitude, pulsed warning vocalizations by a dark male are also unusual.

The major objective of this study was to observe flehmen behavior and investigate its relationship to VNO anatomy. Given that giraffes breed throughout the year, the older, larger males need to be testing the females efficiently to locate individuals coming into estrus; they need an unambiguous testing method for identifying females of interest. Testing urine on the ground would be risky for a bull giraffe with such a heavy head and long neck, and the urine could also be difficult to link with a specific female. Solely investigating the genitalia and urine directly from the female would be the safest strategy for a bull giraffe and is made more necessary by their heavy anatomy. With serendipitous opportunities, we are presenting here additional and more infrequent behaviors of giraffes.

## 2. Materials and Methods

### 2.1. Behavioral Observations

Observations were conducted by pairs of observers in vehicles using spotting scopes and binoculars, primarily at certain waterholes: Klein Namutoni and Chudob, and occasionally at Kalheuwel, Groot Okevi, or Klein Okevi, near Namutoni in Etosha National Park. Observations were taken from 24 May to 12 June 1994 by four pairs of observers and recorders, also from 27 to 31 May 2002 and from 6 to 9 July 2004 by one pair of observers and recorders. A total of 153 h of observations were conducted at the waterholes by pairs of observers. Groups of giraffes, including from two to around twenty giraffes, commonly gathered at the waterhole and in the immediate surrounding area. Observers scanned the giraffes at waterholes for incidents of males inviting or prodding females to urinate, along with the successive outcomes.

Incidental observations were also made. Osteophagia by multiple giraffes was seen during most days of field observatio. A pair of male lions were also observed with a freshly dead, very dark giraffe, which was most likely killed by the lions; observations of this site were made over the following week. Furthermore, in 2002, recordings of pulsed warning vocalizations by a very dark male giraffe were made using a Sony PCM-R300 DAT recorder (Tokyo, Japan) and were subsequently analyzed using Cool Edit Pro (v. 2.1, Syntrillium Software Company, Scottsdale, AZ, USA) to reduce background noise, as well as Spectra Pro (Pioneer Hill Software LLC, Sequim, WA, USA) for analyses.

### 2.2. Anatomical Observations of Vomeronasal Organ

An anatomical specimen of a fetal giraffe was provided by the National Museum of Zimbabwe. Subsequently, portions of formalin-fixed giraffe snouts were provided by the Namibia Ministry of Environment and Tourism.

## 3. Results

### 3.1. Behavioral Observations

**Figure 1:**
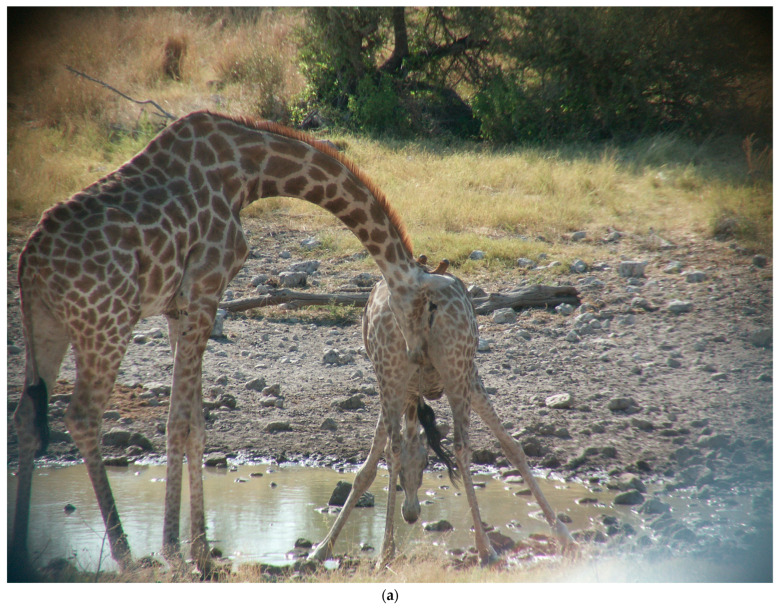

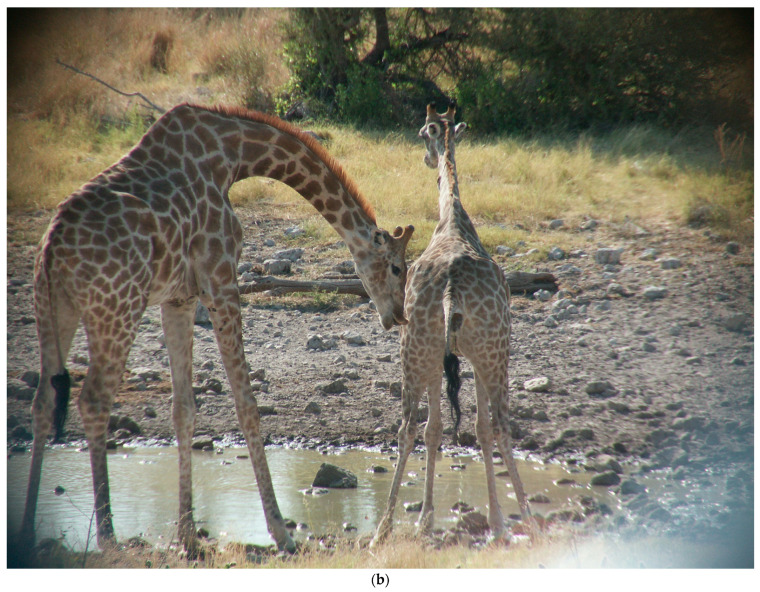

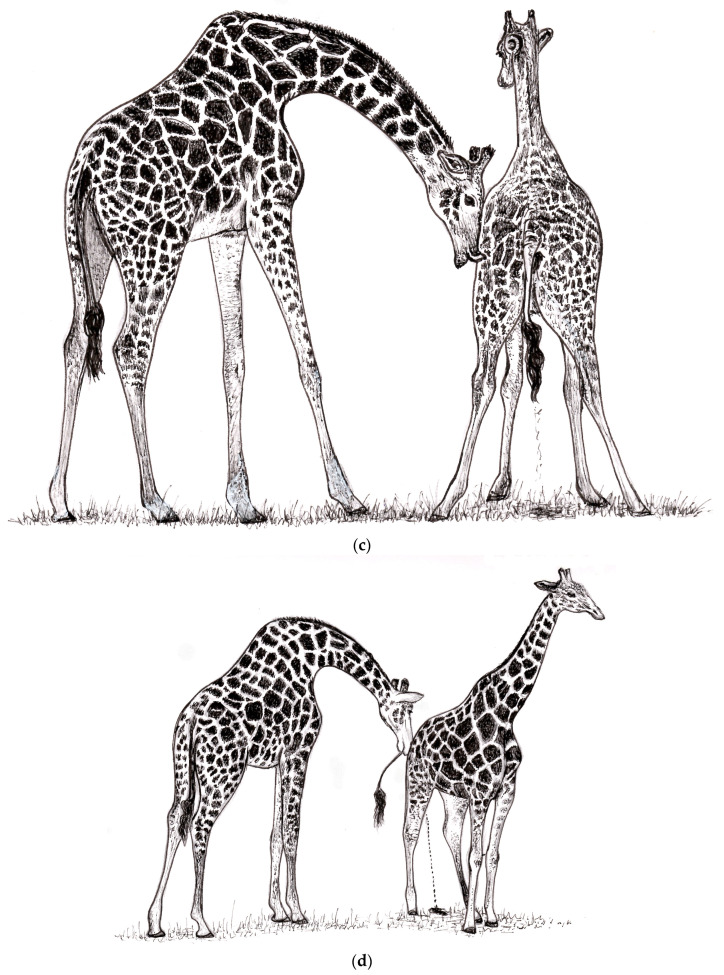
(**a**) The female is drinking at the waterhole, adopting a narrow hind-legged stance, as a male invites her to urinate. (**b**) The female widens and braces her hind leg stance, prior to urinating. She is still urinating, and the male is already beginning his flehmen response. (**c**) An artist’s drawing of Figure 1b. The female has adopted a highly braced hindleg posture and is still urinating. The male has already begun his flehmen episode, even before raising his head. (**d**) An artist’s drawing from a photo of another female in a braced posture while urinating, as the male investigates.

The male giraffe first invites the female to urinate. If she acceded to his request, she would usually assume a wide hind-legged stance, often having to reset her posture, and was well-braced prior to urinating (Figure 1a–d). The male’s duration of sniffing, licking, and investigating the female’s genitalia to provoke her urination was highly variable, ranging from 1 to 10 or more seconds, and occasionally included multiple bouts of investigation of the same female. The female’s urination sometimes began while the male was investigating her genitalia; it persisted for several seconds, allowing plenty of time for the at-hand male to acquire a supply of urine using his tongue and muzzle. The duration of flehmen typically ranged from 3–9 s. His flehmen response often began while his muzzle was still near the urine stream, but he would usually raise his head during most of the flehmen behavior. Males were never seen to investigate urine on the ground.

During the observations on giraffe behavior at water holes where both adult females and males could be observed, the process of a male investigating a female’s genitalia was observed 102 times. The sequence typically began with the male following the female and even nudging her rump. Often, this behavior of the male resulted in provoked urination by the female. The provoking behavior of the males led to 54 female urination events, each lasting around 5 s. When a male was following a female and she urinated, the male placed his open muzzle, including the tongue, in the urine stream, where a little urine was taken into the oral cavity. Following the urination, the males then performed flehmen. For 51 of the urinations, flehmen responses were recorded in the males. Seven other episodes involved an obviously pregnant female and yielded only one urination and one flehmen response. Four pairs were both very young, yielding a single urination and flehmen. Eight pairs involved a very young male and an older female, yielding a single urination and flehmen. Finally, eight pairs involving a very young female yielded no urination but did yield one flehmen from a male investigating the genitalia. Frequently, a very dark male would serially test a string of females in rapid succession—some of them being quite young and refusing the overtures.

**Figure 2:**
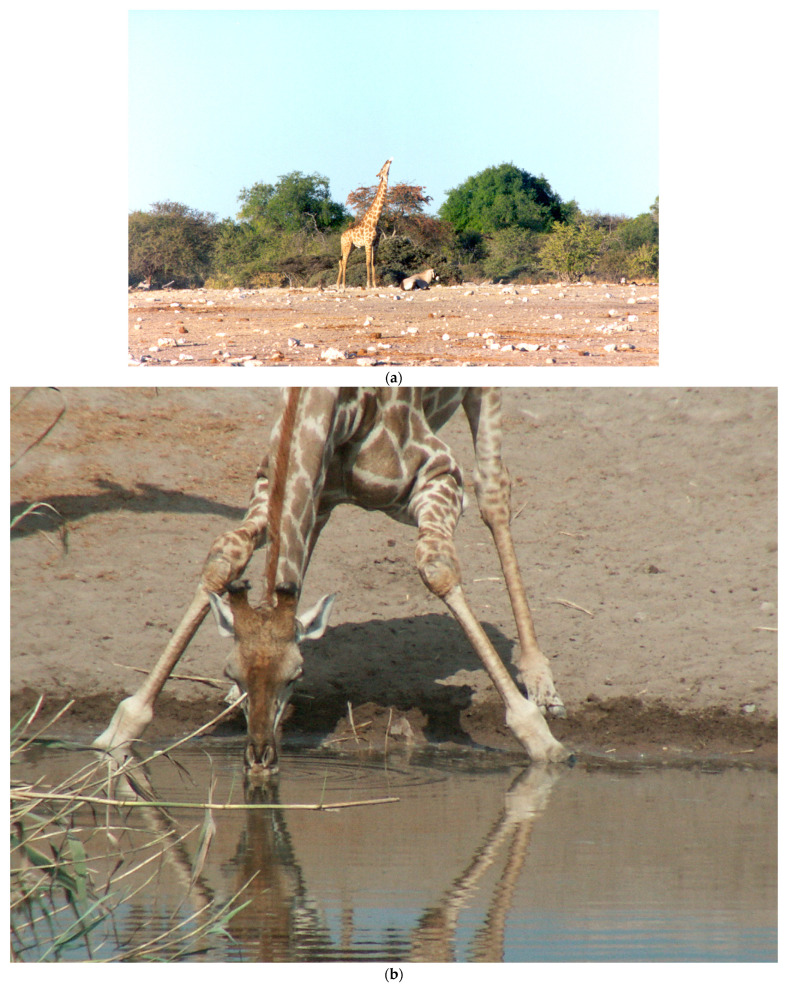

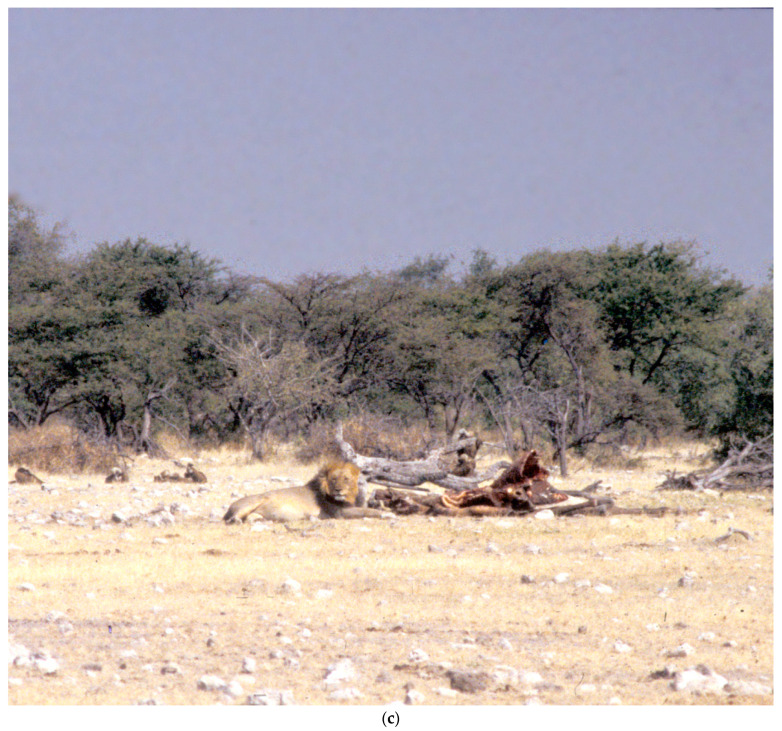

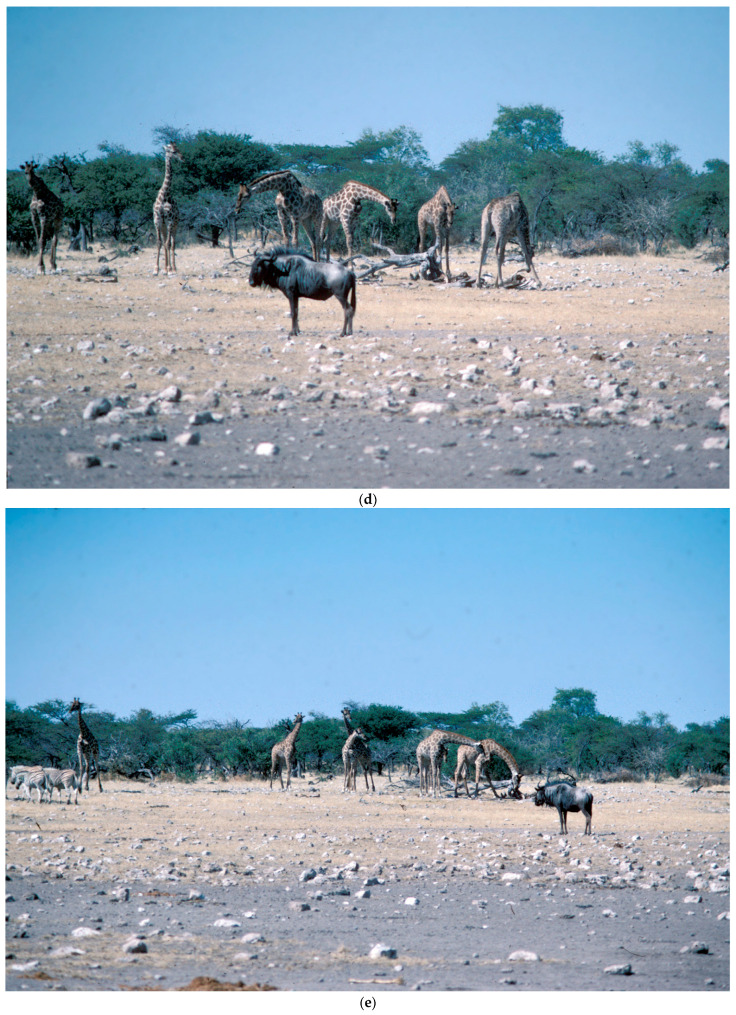

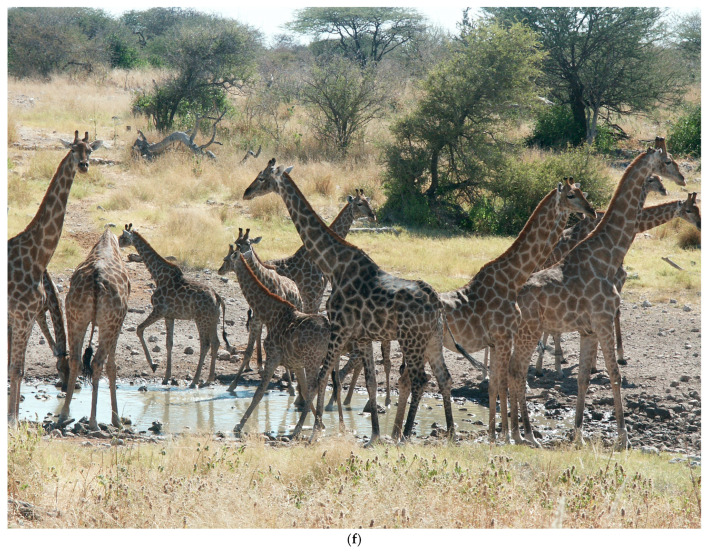

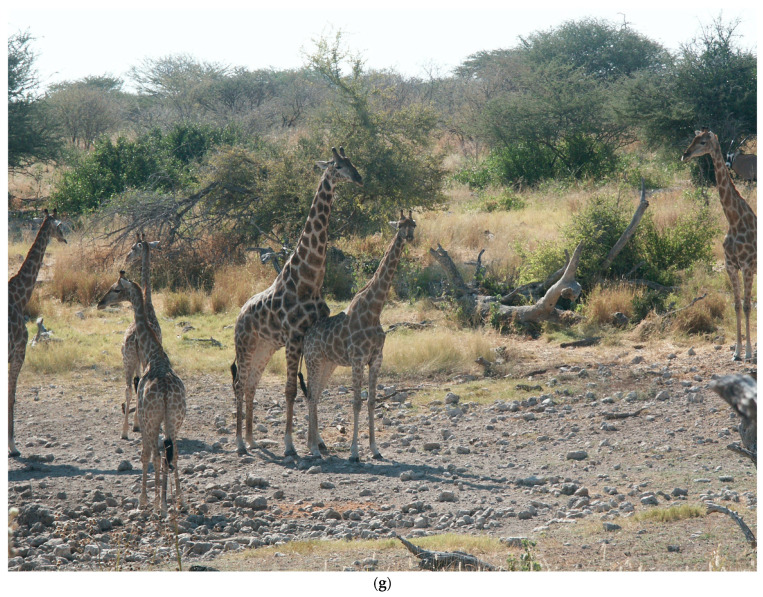
(**a**) Giraffes who are carrying a bone in their mouths often have their heads high in the air, with saliva streaming. (**b**) Drinking at a waterhole puts a giraffe at risk of predation; it could easily be knocked over or fall. As noted by Brand, drinking challenges for giraffes are perhaps responsible for the neck being in an uncommon anatomical position in its junction with the thorax, thus making it possible for the giraffe to breathe while drinking with its forelimbs abducted [10]. (**c**) A lion remaining at the site of the carcass of a very dark giraffe (off to the right), the day after two lions were seen near what appeared to be a fresh kill. (**d**) For five successive days after the lion abandoned the carcass, a steady stream of giraffes passed by the carcass, investigating it for quite a while. (**e**) The giraffes showed great interest in the giraffe carcass. (**f**) A crowd of giraffes on 29 May 2002. These giraffes fled when the bull vocalized, except for one courting pair. (**g**) The proud posture of the male giraffe during a mating episode with attempted mounts; there are many silent onlookers.

The osteophagia practice of a giraffe searching for bones and then chewing them, often with the head high in the air, was seen frequently, especially on the broad plains at the two primary waterholes (Figure 2a). It was seen on most days of field observations. Giraffes would appear to be searching the ground for bones, sometimes in a group. Sometimes a giraffe was seen with a bone stuck in and bulging in its mouth, and the giraffe could not get rid of it. On three separate occasions, an animal appeared stressed and uncomfortable due to a bone stuck in its mouth. Twice, a female with a bone stuck in her mouth was trying to drink and could not do so. One of these females was standing by the water for an hour, walking into the water and slipping once, but not drinking; she had prominent pelvic bones and a large facial bulge on the right side of her face from a lodged bone. One of these females declined to respond to invitations to urinate from two separate males. The other female was chewing and drooling, as well as tossing her head, trying to get rid of the bone; she could not do so and was unable to drink. Giraffes were occasionally seen to slip and nearly fall, reflecting the evident challenges of drinking at the waterhole (Figure 2b); none was seen to actually fall. Another male had a pelvis stuck on his muzzle and was unable to dislodge it; he was still struggling the following day. Sometimes one giraffe tried to grab a bone from another giraffe that had possession of it. One male even peered into the mouth of a male that had a bone in it. Another young male tried to grab a bone from a female. The extensive time that was invested in searching for bones and chewing them diminished the browsing time for those individuals.

The risks of giraffes being preyed upon were demonstrated on 1 June 1994; two male lions were seen, apparently resting by a fresh kill of a very dark-colored giraffe. They were located about 200 m from the road, at the Chudob waterhole. The next day, 2 June, one lion was still sitting on the carcass, sometimes surrounded by 6 jackals and 12 lappet-faced and white-backed vultures (Figure 2c–e). Returning to the site on 5 June, many giraffes were investigating the carcass, as if in a steady and continuing procession. Two vultures were also present. On 6 June, about 15 giraffes were investigating the carcass. On 7 June, the carcass was investigated for at least 1½ hours by 5 giraffes; ribs with vertebrae, skin, and 2 legs could still be seen. The giraffes deviated from their path to inspect the carcass before going to drink. One young male picked up a leg bone from the carcass. By 8 June, no further investigation of the carcass by the giraffes was seen.

During a bout of extensive necking by young adult males in a crowd of giraffes, on 29 May 2002, a mating sequence was also occurring that had been underway for 23 min, culminating in an attempted mount, with other animals freezing in place. Then, another very dark male on the sideline gave a high-amplitude growl, consisting of three pulses of sound with a 30.6-second duration, at which point the giraffes fled the waterhole. The entire giraffe herd began to gallop away, except for the mating pair. The dark male continued vocalizing while the animals were running away. The attempted mounting continued, and several giraffes came into the area, comprising a group of 22 giraffes (Figure 2f,g). All watched the mating sequence. About 45 min later, the young males were sparring again. Again, a very large and dark male growled, with 12 pulses of over 105.9 s; immediately, the herd galloped away, except for the two sparring males. Four of the pulsed vocalizations were analyzed and all included a formant frequency near 209 Hz, with an average duration of 0.33 s.

Contentious activity also arose on 8 July 2004, with considerable inter-male conflict. One male demonstrated fresh chest wounds. Another male with bloody hip wounds drove off another male, which was also snorting.

### 3.2. Anatomical Observations of the Vomeronasal Organ

**Figure 3:**
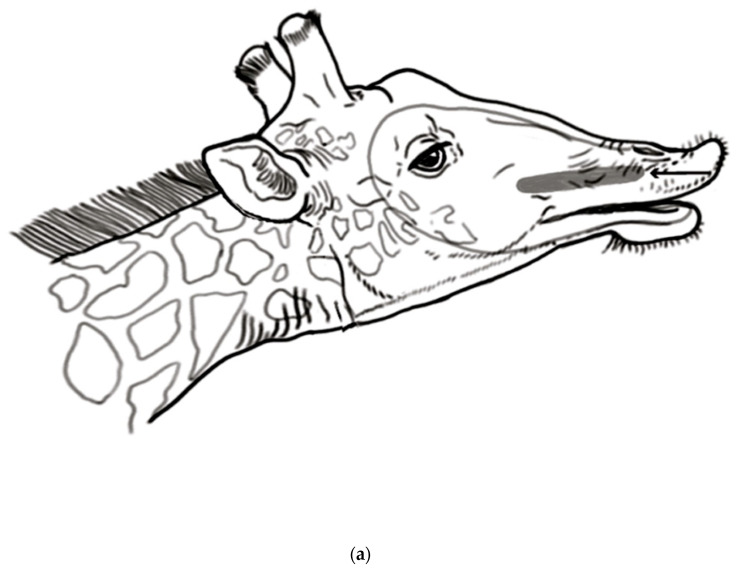

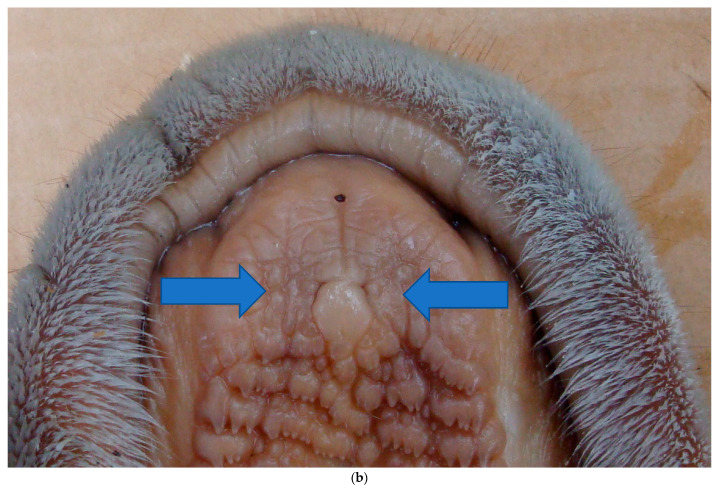

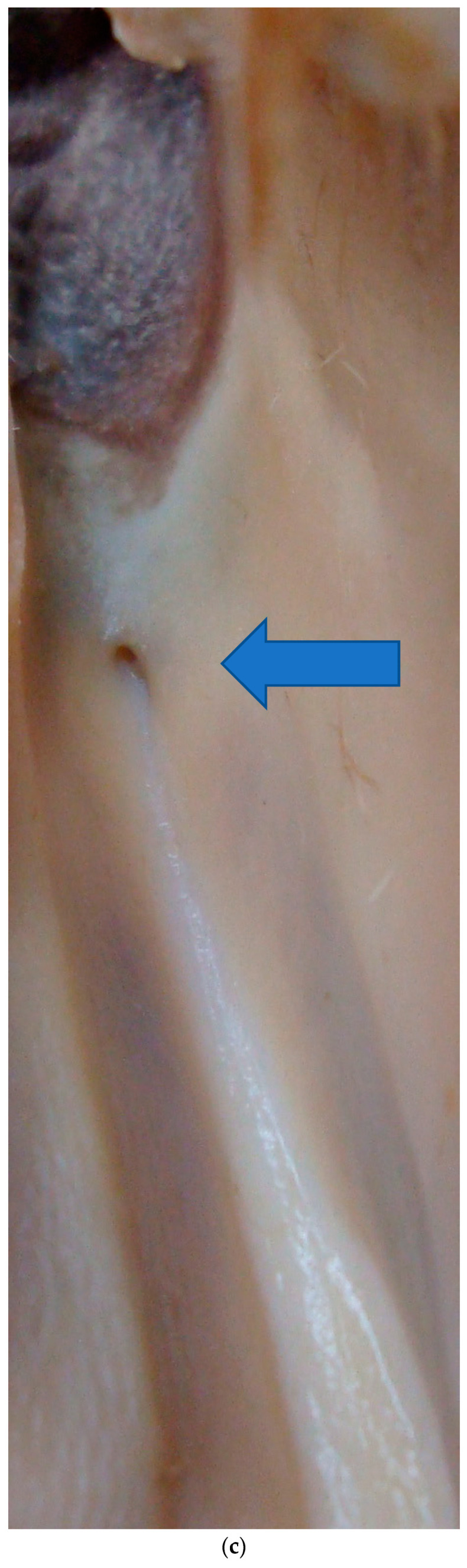
(**a**) Schematic diagram showing the route (in dark gray, indicated by an arrow) of the olfactory stimuli as urine passes through the incisive papilla to the incisive duct and into the tubular VNO on the right side. (**b**) The incisive papillae of the giraffe, indicated by the blue arrows, connect to the incisive ducts leading to the VNO. (**c**) The blue arrow indicates the small opening of the olfactory incisive duct, leading to the vomeronasal opening, which lacks a pronounced funnel.

In typical ruminants, the incisive papillae provide access to the bilateral tubular VNOs from the oral cavity, as schematically depicted in Figure 3a. In the two giraffe specimens examined, the incisive papillae were prominent. The opening of each incisive duct, leading to the VNO, was apparent (Figure 3b). In contrast to the usual artiodactyl, funnel-shaped nasal opening to the VNO, the dissection of the nasal cavity revealed that there was what appeared to be only a very small nasal duct (Figure 3c). The vomeronasal anatomy of the giraffe is more specialized toward connecting with the oral cavity than with the nasal cavity.

## 4. Discussion

The physical structure of the giraffe, with its elongated neck and legs, obviously serves this species very well, allowing them foraging access to highly placed leaves in tall trees. Giraffes have acquired the behavior of splaying the front legs, allowing them access to water, despite being capable of doing without it for long peiods [10]. The observations reported here, although limited, suggest behavioral and anatomical adaptations to their physical structure to enable the investigation of urinary pheromones that indicate impending estrus in females.

The behavioral adaptation is that a male needs to be vigilant to catch and test female urine while it is being expelled into his mouth for delivery into his VNO. Standing behind a female, perhaps nuzzling or tapping her rump, often triggers urination. Females that are near estrus will wait until a male is near to urinate.

Comparisons with antelopes, which we have studied previously, are interesting. Although oral connections to the VNO were missing or were extremely limited for alcelaphine antelopes, nasal connections were observed (resembling a funnel) between the ventro-rostral end of the nasal cavities and the VNO in all antelopes studied, comprising both non-alcelaphines and alcelaphines, including Thomson’s gazelle, Grant’s gazelle, impala, eland, black wildebeest, blue wildebeest, topi, Hunter’s hartebeest, blesbok, and Coke’s hartebeest [18].

An adaptation in gross anatomy means that the access to the VNO for the giraffe is primarily through the bilateral incisive ducts, with a much more limited access through the nasal cavity. This VNO connectivity differs from antelopes, such as gazelles, impala, and eland, which have pronounced oral and nasal connections to the VNO [18]. This proposed adaptation is consistent with a secondary function of the VNO in East African ungulates, which was previously proposed to be intended for detecting the vegetative olfactory cues that indicate season [18]. Those other ungulates have reproductive cycles that are tuned to weather patterns and seasons each year. In contrast, giraffes have a longer gestation of 448 days [23], which is not locked into a certain time of year.

For the female giraffe, consorting with the senior males that are so much larger and have large heads can impose some risks. She must stabilize herself, even at the early stage of courting, by widening her stance and bracing her legs. The fact that male giraffes never access urine from the ground resembles the behavior of the large antelope, the eland; for 90% of the time, the male tests the female’s readiness by either using the urine stream or the female’s genitalia [19]. For such large animals as the eland or giraffe, reaching the ground necessitates great effort and is risky.

As emphasized by Brand [10], the arid environment of Etosha National Park, especially in the dry seasons, combines with the waterholes to favor large dark male giraffes in terms of their mating success. In our study, these conditions meant that more giraffes were present for much of the time; a higher density of giraffes can also lead to more intense behaviors than are typical—as sometimes occurred in these observations. This helps explain the greater frequency of some of the more unusual behaviors described here. For example, although giraffes were long thought to be mute, they can emit loud warning calls that are sufficient to affect the behavior of other giraffes and cause them to flee. The procession of giraffes investigating the carcass of the dead giraffe is mysterious; were they exploring and expressing reverence for a familiar animal, or prospecting for bones to chew?

Their body size, use of space, and other unique characteristics of giraffes make them challenging to study and understand. This project touches on some additional uniqueness in terms of the coordinating reproductive behavior demonstrated between the male and female giraffes.

## 5. Conclusions

Among the ungulates, giraffes stand alone in their ability to breed throughout the year. In coordinating their reproductive behavior, they employ their own style. The male must figure out which female is coming into estrus soon, and he must gain her cooperation when he seeks to assess her current status. By cooperating and urinating, if she is not in proestrus, she can be left alone after one encounter. If she is in proestrus, she has an opportunity to make her decision, affecting mate choice.

## Data Availability

Research data from field records are available from the authors. No human subjects were involved in this study.
